# Encyclopedia Americana: Supplementary Volume

**Published:** 1847-08

**Authors:** 


### PART II.—REVIEWS.

ARTICLE VIII.

*Encyclopedia Americana:* Supplementary Volume. A popular Dictionary of Arts, Sciences, Literature, History, Politics, and Biography. Vol. xiv. Edited by HENRY VETHAKE, L.L.D., Vice President and Professor of Mathematics in the University of Pennsylvania, Member of the American Philosophical Society, Author of a Treatise on Political Economy, etc. Phila.: Lea and Blanchard. 1847. pp. 663, 8vo. (From the Publishers—For sale by Joseph Keen, Jr., Chicago, Illinois.)

The book which we propose to introduce to our readers in this notice, is a supplementary volume of the Encyclopedia Americana; a work which has been before the public for fourteen years, and has been considered worthy of a place, among works of reference, in almost every library of any pretensions. It was founded on the basis of the "Conversations Lexikon," a German work, of high repute and which has passed to a ninth edition, now in course of publication. The rapid growth of the arts and sciences, and the many subjects of interest in the other departments of which the encyclopedia professes to treat, which have arisen or extended their limits within the last fourteen years, have left the original work so far behind the age, that the present volume was imperatively called for "to restore to the work all the advantages which belonged to it originally, as a book for ready consultation on subjects of general or popular interest."

The editor, Professor Vethake, has been long sufficiently known to the literary and scientific of our age, both as a teacher and author; but we are happy to have this opportunity afforded us to offer our small mite of admiration of his high worth and excellence as an erudite scholar, and of esteem for his many excellent qualities of a social character, which we have had an opportunity of learning, not only by perusal of his writings, but by personal acquaintance with him in the walks of private life. Indeed, we may safely assert that we know of no one better fitted for the task which he has assumed and accomplished; a task demanding for its perfect completion, discrimination in selection of material, judgment in arrangement, and much research and information of a diffuse and general character.

In the biographical article, Professor Vethake has selected with much judgment the names of those citizens of *foreign* countries who enjoy high reputatation; but has judiciously abstained from notice of any Americans of distinction, but such as have been removed by the hand of death from the field of envy, detraction and prejudice, and to whose memory justice impartial may now be done. In perusing the biographical sketches of living foreigners, eminent for literary and scientific abilities, we have been gratified to learn much that we would not know where else to find, and we have been particularly delighted with the notices of the recent deceased of our own times and country, whose biographies have now for the first time been presented to the world.

The historical and political articles are brought up to the present date, and will be read with interest by all. Their conciseness and clearness, together with the mass of statistical information which they contain in their connection with commerce and population, render them highly valuable.

But it is in the scientific department of the work that our readers will be more particularly interested, and to this we purpose to give a more extended notice. As, however, our space will not permit a notice of many subjects, we have selected one article which is of most length, and in which we think, considering the condensation which the work required, much justice has been done to the subject, itself of considerable interest to our professional and scientific readers. The article we refer to is that on "Magnetism." In the original work, electricity, galvanism, and magnetism were treated of separately, but in the present volume they are all treated under the one heading; the intimate connection which comparatively recent researches have shown to exist between these forces, rendering it highly advantageous to treat of them in this way. The order in which the article presents these subjects is as follows:

I. *Ordinary Electricity;*
II. *Galvanism;*
III. *Magnetism;*
IV. *Electro-Magnetism;*
V. *Thermo-Electro-Magnetism;*
VI. *Magnetic Electricity; and*
VII. *Animal Electricity.*

Upon these various subjects the article before us reviews all that has been done in the last fourteen years, and is the most complete and condensed account of the advance of electrical science that we have yet met. The matter which it contains, indeed, is only to be found in monographs of the discoverers, distributed through the various foreign and American scientific publications of the day. These have had their contents sifted, and in the article under review, is presented the pith of them all, as far as the limits of a work of the kind would permit. We will lay before our readers a condensed abstract of the various headings just enumerated, endeavoring, if possible, to include all the recent discoveries of importance.

"By *Ordinary Electricity* we understand that which is usually evolved by means of friction in the common electrical machine, and which differs from galvanism in the greater *intensity* of its action, and in the smaller quantity of the agent operative in the production of a given phenomenon."

*Velocity of Electricity.*—This important discovery the world owes to Professor Wheatson of King's College, London, and to give a satisfactory view of the ingenuous mode by which the inconceivably rapid passage of the electrical current, was measured, we copy at length the account given in the work before us.

The problem consisted in determining the velocity of transmission of an electrical discharge from a Leyden jar, through a long conductor.

"A copper wire, some fifteenth of an inch in diameter, and half a mile long, was insulated in such a manner that its parts were not in contact with each other, three breaks being made in it; one near the beginning, another near the end, and the third near the middle of the length.

"These breaks were then brought over each other and arranged, say in a vertical line, before a small mirror which could be made to revolve, by means of watch work, eight hundred times in a second. When the mirror was at rest, and a charge of electricity from the jar was passed through the long wire, three sparks were seen in the reflector apparently at the same instant, one above the other, as shown in the first figure in the margin. But when the mirror was made to revolve at its full speed, the appearance was that exhibited by the second figure. 1st. Each spark appeared elongated or drawn out into the appearance of a line of light, indicating that the duration of the discharge occupied an appreciable portion of time; for if the spark were not absolutely instantaneous but required sometime to pass the opening in the wire, and the mirror revolved sufficiently fast, the light would be reflected at each instant in a new direction; and, on account of the continuance of the impression on the eye would exhibit a line of light. 2d. The spark at the beginning of the wire was vertically over that at the ending, while in all the experiments the spark at the middle was thrown a little to one side: this appearance proves that the disturbance of the electrical equilibrium commences simulteneously from each end of the wire, and arrives last at the middle. By measuring the distance of the eye from the mirror, and the apparent lateral deviation of the middle spark from the other two, the fractional part of the revolution of the mirror performed during the passage of the discharge from either end to the middle, or through half the length of the wire, could be obtained; and knowing the length of the wire and number of revolutions of the mirror in a second, the velocity of the dis charge could be determined. In this way, Professor Wheatstone found the velocity of the discharge to be about two hundred and eighty-eight thousand miles per second, ro greater than that of light through the celestial space. He also inferred from the elongation of the light, that the spark though not absolutely instantaneous, occupied in its passage less than the millionth part of a second. The fact that the discharge from a Leyden jar reaches the middle of a long wire last, has been thought to favour the hypothesis of two fluids passing in opposite directions; but the same fact is also a legitimate consequence of the hypothesis of a single fluid."

Fig. 1.

Fig. 2.

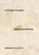

*Specific Inductive Capacity.*—Under this head is presented the views which Dr. Faraday professes to have established by conclusive experiments, relative to the different powers possessed by different substances, of transmission of inductive influence. Dr. F. ascribes induced electrical excitement to a polarization of the particles of the non-conducting medium interposed between the excited body and the body in which inductive excitement is produced. Differences in inductive capacity were found to exist among liquids and solids, but all gaseous bodies possessed the same capacity, which was not altered by variatiations of density, elasticity, dampness, or dryness. Dr. F. assumes the fact that induction takes place in curved as well as straight lines, which our author thinks he is not warranted in doing.

"Dr. Faraday has also made a series of investigations, relative to the discharge of ordinary electricity. These he has classed under three heads, namely, the discharges by *conduction, disruption, and connection.* The *discharge by conduction*, takes place without chemical action, or any necessary displacement of the particles of the conductor; but different bodies oppose very different degrees of resistance to this action. He finds that all bodies except gasses are, to a certain extent conductors; that all oppose a certain amount of resistance to the passage of the electrical discharge; and that conduction and insulation are different degrees of the same quality. The *disruptive discharge* is that which takes place generally between the conductors in the form of a spark. It varies very much when different aeriform substances are interposed between two conductors. An apparatus was constructed in which the same discharge could pass through either of two cylinders, one filled with gas submitted to the experiment and the other filled with common air used as a standard of comparison. With this it was found that muriatic acid gas has nearly three times the specific insulating power of hydrogen, and nearly twice that of atmospheric air. The disruptive discharge sometimes changes its form from the spark to the brush, and the latter is shown by Professor Wheatstone, to consist of a succession of intermitting discharges. The continued sound which accompanies the brush, is due to the recurrence of the souud from each discharge: the brush exhibits specifie characters in different media, which are manifested by difference of colour, light, form, and sound. The *connective discharge* is that produced by the motion of charged particles. The particles of air, for example, in contact with the projecting part of a conductor, become highly electrified and are repelled by the conductor, thus giving rise to currents of air which rapidly carry off the charge. Dr. Faraday traces an analogy between this discharge and the galvanic current, in which, as has been shown by Ampere, the consecutive portions repel each other."

*Electricity evolved from a Steam-Boiler.*—Most of our readers are perhaps aware that the most powerful electrical machine ever exhibited in this country, was constructed of a peculiar arrangement of a steam boiler. The article before us gives an account of the manner in which electrical excitement may be produced by this means. We accordingly extract it:

"In 1840 a workman near New Castle, in England, discovered that, while one of his hands was plunged in a current of steam issuing from a boiler, a spark would pass between his other hand and any conductor to which it was approached. We owe to Dr. Faraday the discovery of the proper explanation of this phenomenon. He has shown by a set of conclusive experiments, that the evolution of the electricity in this case is due to the friction of the drops of water from the condensed vapor against the sides of the tube, through which they are impelled by the elastic force of the steam. If the current issue through a stop cock in the side of the boiler, it produces at first, or so long as the cock is cold, a powerful excitement; but the moment the metal becomes so warm as not to condense the steam, the electricity disappears. Any substance that increases the conducting power of the liquid, being placed within a hollow in the stop-cock, destroys the existing power as effectually as moisture does that of the rubber of the machine. The final conclusion from the researches made, is, that pure steam or gases do not excite electricity by friction against solids or liquids, and that the effect, in all cases, is due to the presence of a liquid which rubs against a solid. Mr. Armstrong, availing himself of this method of developing electricity, has constructed an electrical machine of immense power, which consists principally of a tubular boiler, about the size of that of an ordinary locomotive, insulated on stout glass pillars, and furnished with a series of pipes through which the steam is blown off. One of the largest instruments of this kind has recently been exhibited in this country."

*Dynamic Induction of Ordinary Electricity.*—The laws governing the phenomena of dynamic induction of ordinary electricity, having been entirely developed by a philosopher of our own country—Professor Henry, of Princeton, New Jersey—and the phénomena being themselves of a novel and highly interesting character, we feel ourselves warranted in presuming them of sufficient interest to our readers to give them more space than other departments of the subject. It having been our great privilege to have witnessed a portion of the experiments conducting the professor to his generalizations while serving him in the humble, but much valued capacity of assistant, we can bear witness to the almost incredible amount of severe thought and physical labor expended upon the investigation. Having thus become aware of the great scientific value of the discoveries, it has pained us that while credit has been given to Professor H. abroad, his valuable additions to science have been so little appreciated in his own country, and that the knowledge of the important developments effected by him should have been so little diffused. Justice, though tardy, is now, we are happy to say, about to be rendered; and government by appointment, to an office of high trust, and demanding great scientific attainments, has begun to redeem itself from the odium of neglect of a distinguished individual, whose name will be prominent in the scientific history of our country, long after his political cotemporaries will have sunk into oblivion. We give in full that portion of the article devoted to this branch of the subject.

"In 1836, Professor Henry, of Princeton, commenced a series of investigations, the object of which was to ascertain whether there existed any dynamic phenomena in ordinary electricity analagous to those found in galvanism. His labors on this subject, which have been continued at intervals up to the present time, have given rise to a series of new phenomena, of which our limits will not permit us to give more than the following brief analysis:

"1st. When the discharge of a Leyden jar is transmitted through a conductor, for example, a copper wire, it induces in an adjoining parallel wire, a current of electricity analagous to that which, under similar circumstances, is developed by a galvanic current.

"2d. The direction of this induced current, as indicated by the magnetic polarity given to a steel needle, enclosed in a spiral forming part of the circuit, changes its sign, (1) with a change in the distance of the two wires; (2) with the proximity of a third parallel wire, formed a closed circuit; (3) with an opening in the wire which receives the induction; and, (4), with the quantity and intensity of the discharge.

3. When the first induced current is made to act on a third wire, a second induced current is produced, which, in its turn may give rise to another current, and so on. When the several wires are at a considerable distance from each other, and the direction of the several currents is determined by the magnetization given to a sewing needle, we have, (in calling the direction of the current from the jar plus,)

$$+-, +-, +-,$$

&c.; that is, each succeeding current is opposite in direction to the current which induced it.

"4th. When a plate of metal is placed between two flat spirals, and a discharge from a jar passed through one of them, the induced current which would be produced in the other, is neutralized by an adverse current induced in the plate.

"5th. The dynamic induction of ordinary electricity takes place at a surprising great distance. A discharge from a jar being passed through a parallellogram of wire, arranged around the ceiling of an upper room, magnetized needles in a spiral forming part of a corresponding parallellogram placed in the floor of the cellar, at the distance of thirty feet below. In another set of experiments, needles were magnetized in a parallel wire at the distance of three hundred feet from the primary current; and from all the experiments it appears that the inductive results may be obtained at an indefinite distance, provided the length of the parallel wires be increased in proportion to the distances to which they are separated.

"6th. Inductive effects similar to those we have described can also be obtained from the discharge of the thunder cloud. Professor Henry attached to the metallic roof of his house a copper wire, which, passing through his study, terminated in a deep well near by: a needle placed in a spiral forming a part of the conductor through the study, was magnetized at every flash of lightning within a circle of twenty miles in diameter around Princeton. The needle is sometimes magnetized in one direction and sometimes in the other.

"7th. When sparks are thrown from a small machine on the middle of a lightning rod, the electricity does not tend to pass silently into the earth; on the contrary a spark can be drawn from every part of the rod, even from that near the earth.

"8th. It is also proved by the magnetization of a needle placed inside and outside of a hollow bar, that although galvanic electricity passes through the whole capacity of a conductor, ordinary electricity passes principally at the surface. It is also shown, by a series of conclusive experiments that, during the first moment of the discharge of a Leyden jar, half the length of the conductor next the end at which the discharge enters is *plus*, and the other half is *minus*, the intensity diminishing each way to the centre; and that at the next moment the condition is reversed, the opposite end becoming *plus* and the other *minus*.

"After a laborious investigation of the phenomena we have described, Professor Henry has succeeded in referring them all to the hypothesis of the existence of an electrical plenum through which dynamic induction is transmitted wave fashion; and in showing that, in all cases of the disturbances of the equilibrium, the fluid comes to rest by a series of oscillations. Thus, in the discharge of the jar, all the phenomena indicate a principal action in one direction and a series of reflex actions backwards and forwards, each more feeble than the preceding, until the equilibrium is restored.

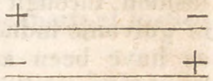

"Our limits, however, will not permit us to give in detail the application of this hypothesis to the explanation of this phenomena; we can only allude to the explanation of a few of the more prominent ones. In the discharge of a Leyden jar through a long wire, we know that the disturbance reaches the middle of the length last; and this may be explained by supposing a quantity of free electricity to enter the end of the wire next the knob, and, at the same time, an equal quantity of the natural electricity of the wire to be drawn from the other end next the outside. The whole wire, for an instant, must therefore be thrown into a state represented by the upper line in the annexed diagram, in which the first end is *plus* and the other *minus*. The natural electricity of an adjacent parallel wire will, according to the law of ordinary induction, assume for a moment the condition represented by the second line, and in passing to this state from that of equilibrium, it will exhibit a current adverse to that in the primary wire; but the electricity of the primary wire will pass to its ordinary state of equilibrium by a series of oscillations, each of which will induce an opposite wave in the second wire. If the two two wires be at such a distance from each other, that the induction of only the first wave is effective in giving magnetism to the needle, it is evident from theforegoing that we shall always have a polarity indicating an induced current opposite to that of the primary current; but if the wires be brought nearer each other, then the needle will be first magnetized to saturation by the first vibration; and immediately afterwards the magnetism will be discharged and that of an opposite kind developed by the second vibration. In the same way, if the wire be placed still nearer, another charge will be produced in the polarity of the needle due to the third vibration, and so on.

"It is not difficult to perceive that the variation in other conditions may also change the polarity of the needle, thus: suppose a break be made in the circuit of the secondary current, then the primary wave, having the greatest intensity will alone pass through the opening, and the polarity of the needle will be due to the action of this; while if the opening be closed, the residual magnetism of the needle may be due to the action of some of the succeeding vibrations. In this way all the phenomena described in (2) are readily explained.

"Also all the phenomena of the lateral discharge (7) find an easy explanation in a reference to the same principles. At the moment a charge is passing along the wire, each consecutive part of the length of the conductor becomes charged in succession, and tends to give off a spark to all neighboring conductors, for the same reason that a conductor charged with statical electricity tends to produce the same result. All the phenomena of dynamic induction are analagous to those of statical induction, with this exception, that, in the former, we are obliged, in order to explain all the facts, to admit *time* as an element of the calculation. The indefinitely greater distance at which dynamic induction takes place between two parallel wires, than that at which statical induction would be exhibited under the same circumstances, together with other phenomena, lead us to suppose that the former induction is transmitted in time, wave fashion, through the electrical plenum. All the phenomena of galvanic induction may be referred to the same principles as have been adopted in the explanation of the facts we have given in this article."

II. *Galvanism.*—Under this heading the improvements in the construction of the galvanic battery are mentioned, and the objects gained by those improvements explained. As these may, however, be found sufficiently and indeed more fully detailed in recent works on chemical philosophy, (see Daniels' Introduction to Chemical Philosophy and Graham's Chemistry,) we prefer omitting it, as also the article on *electro-chemistry*, that we may be able to give space to a few other extracts upon subjects not to be found in ordinary and easily accessible works.

III. *Ordinary Magnetism.*—Upon this branch of the subject our author gives an account of the experiments simultaneously made by a number of observers, and in different parts of the world, with a view to ascertain the *declination* or *variation*, the *inclination* or *dip*, and the *intensity* of magnetism. As the connection of these subjects with the objects of our Journal are very remote we omit them entirely and pass on to—

IV. *Electro-Magnetism.*—As the facts upon this subject are generally known, we shall not stop to detail them, but merely state that credit is given by Professor Vethake to Professor Henry, of Princeton, for the most valuable recent additions to this department of science, and accords to him the merit of first producing motion by a combination of the forces of magnetic and electrical attraction—in a word of being the inventor of the electro-magnetic machine.

V. *Thermo-Electro-Magnetism.*—The only new fact that we find in this portion of the article which we have not found in ordinary works, is the following:

"Professor Henry has lately made an interesting combination of the thermo-electrical apparatus and the telescope. He places the end of the thermo-electrical pile at the point where the eye-glass is inserted in the tube of a reflecting telescope; and, by this combination, is enabled to detect the difference of radiation from small objects at the distance of several miles. It also exhibits a remarkable difference in radiations from different clouds and also from different parts of the clear sky, and will, therefore be an important instrument for meteorological purposes."

*Magneto-Electricity and Galvanic Induction.*—We know of no work which gives an account of this branch of science at all comparable in clearness and conciseness with the book under review. So comprehensive is it, that we feel that any attempt to condense it would be to leave out portions equally important with others we should retain; and though our article has already expanded, beyond our first intentions, we feel impelled to give the extract entire:

"By magneto-electricity is understood the electricity which is developed by magnetic induction. The first discoveries in this division of our subject, which were made by Dr. Faraday in 1832, may be briefly stated as follows:—1. If a magnet be suddenly approached to a mass of conducting metal, a current of the natural electricity of the conducting substance is produced in the conductor, in a direction parallel and opposite to that of the hypothetical current, which, according to the theory of Ampere, revolves around the magnet at right angles to the axis. 2. So long as the magnet remains at rest near the conductor, no effect is observed; but the moment it is drawn away, a current takes place in the same direction as that of the hypothetical current in the magnet. The current in each case continues only during the time of the motion of the magnet. 3. If a piece of soft iron be suddenly magnetised near a mass of conducting matter, a current of electricity will be induced in the conductors in the same direction as that produced by the approach of a permanent magnet. 4. So long as the magnetism of the iron remains of the same intensity, no current is observed; but if the intensity be suddenly diminished a current is produced in an opposite direction to that of the current which was developed at the time of the magnetizing of the iron. The current, in both cases, continues only during the indefiinitely short period in which the iron is undergoing the change of state. 5. These facts may be readily certified by coiling a long covered wire into the form of a ring, about four inches in diameter and consisting of about one hundred turns. The two ends of the coil being connected with a galvanometer, a current in one direction will be exhibited when a bar magnet is thrust into the axis of the ring, and another in an opposite direction when the bar is drawn out.

While the magnet remains at rest, with its ends for example, just within the ring, no current is indicated; but if it be pushed further in or drawn further out the needle will be deflected by an induced current in the wire. Also a current will be induced in a coil by placing in its axis a bar of soft iron, and suddenly magnetizing this, either by approaching to its two ends the opposite ends of two magnets, or subjecting it to the influence of a galvanic coil, or even to the inductive action of the earth, as is the case of magnetizing a bar by suddenly turning it in the direction of the dipping needle. A magneto-electrical machine, which will produce a rapid succession of alternate currents, may be formed in this way, by mounting a bar of soft iron surrounded by a long coil of wire on an axis like a dipping needle, and causing it to revolve rapidly in the plane of the magnetic meridian; at each half revolution of the bar its polarity is changed, and consequently a series of currents in opposite currents is produced. To exhibit, however, the effect by the mere induction of the earth, a large bar, surrounded by a very long wire, is required. A much more intense induction is produced by causing the ends of the same bar, at each half revolution, to pass near the opposite poles of two permanent magnets, or, what amounts to the same thing, by making them revolve before the two poles of a horse-shoe magnet. Various machines have been devised in accordance with these principles, for exalting the effects and facilitating the experiments. The most complete arrangement now in use was the original combination of our countryman Mr. Joseph Saxton. With a machine of this kind, shocks, decompositions, and all the other effects or galvanism, may be produced.

"*Galvanic Induction.*—From the well-known identity of action of a permanent magnet and a cylindrical coil through which a galvanic current is passing, it might be reasonably supposed that effects similar to those we have just described might be produced by galvanism; and the truth of this inference was also proved by Dr. Faraday. The results obtained by him may be stated as follows:—1. When a conductor, for example, a copper wire, through which a current of galvanism is passing, is approached to another conductor arranged parallel to the first, a current of the natural electricity of the metal is induced in the second wire in a direction contrary to that of the inducing current. 2. As soon as the motion of the conductor ceases the current ceases, and, however long the two conductors remain relatively at rest, no effect will be produced on the needle of the galvanometer; but the moment the inducing current is withdrawn, a reflex current is exhibited. 3. If two conductors are placed near and parallel to each other, and a galvanic current is passed through one of them, a momentary induced current in the opposite direction, will be produced in the other. 4. So long as the galvanic current is kept at the same intensity, no current is observed in the adjoining conductor; but the moment the galvanic current is stopped, a current in the same direction as that of the original currrent is induced in the adjacent wire. 5. All these principles are readily proved experimentally, by substituting, in the experiments we have given under magneto-electricity, instead of the bar magnet, a coil in the form of a cylinder, consisting of several hundred feet of wire, through which it is made to pass. 6. After the discovery of Dr. Faraday of the foregoing principles of galvanic induction, the most important additions to this branch of electricity have been made by Professor Henry. The following is a brief analysis of his results:

"*Induction of a Current on itself.*—When the poles of a single battery terminating in cups of mercury, are joined by a short wire, no spark, or at least a very feeble one, is obtained at the moment of breaking the circuit. But when the same poles are joined with a long wire, a brilliant spark is exhibited each time one end of the wire is drawn from the cup of mercury; and if the conductor be sufficiently long, pungent shocks may be obtained by grasping the end of the wire, while the rupture of the current is made. These effects Professor Henry has shown to be due to a momentary induction in the conductor itself, by which, at the moment of the cessation of the battery current, the natural electricity of the metal is put into intense motion. The effects, which are increased by coiling the conductor on itself, are of two kinds; those of quantity and those of intensity. Those of the first kind are best exhibited by means of a conductor of copper riband, about one hundred feet long and an inch and a half wide, covered with several thicknesses of silk, and wound into plain spiral like the main-spring of a watch. When one of the projecting ends of the coil is fastened to one of the posts of a small single battery, and the other end drawn along a rasp attached to the other pole, a series of deflagrations is produced as brilliant as those excited in the ordinary way with a battery of twenty or thirty pairs highly excited. To produce, however, the most pungent shocks from a small battery of a single pair of plates, the coil should be formed of a conductor four or five hundred feet long. The pungency of the shocks may be much increased by placing in the axis of the coil a bundle of iron wires; we shall then have the inductive effect of the temporary magnetism of the iron added to that of the coil itself. On this principle the machines for medical purposes are now constructed. 7. *Conditions which influence the production of a secondary current.* When a flat riband coil, such as we have described, has been placed on another coil of the same kind, and a current of galvanism of a single battery passed through one of them, an induced current of quantity, or such as will produce brilliant deflagrations and magnetise needles, will be developed in the other. If, in place of the riband coil, which received the induction in the last experiment, there be substitued a coil formed of fifteen hundred yards of fine covered wire, an induced current of intensity will be produced, or such as gives pungent shocks. With a single battery, these effects only are exhibited at the ending of the primary current; but if a compound battery of fifteen or twenty pairs be used, the same results are obtained at the moment of beginning of the battery current. It is, however, a remarkable fact, that with a galvanometer, the quantity of induction, in all cases, appears to be the same at the completion and interruption of the primary circuit. 8. The induction of a secondary current may be produced at a considerable distance, by coiling a long riband conductor into the form of a hoop, three or four feet in diameter, and placing opposite to this a coil of long wire. In this way shocks may be given through the partition walls of two adjoining rooms, when the hoop is suspended on one side, and the long wire coil brought opposite to it on the other. 9. The interposed substance in the last experiment, must consist of some non-conducting material; for if between the two coils a plate of metal be placed, the shock is entirely neutralized; and also, surprising to say, the same result is produced, but less perfectly, if the plate be placed, not between the coils, but on the outside of one of them. This result is shown to be due to an induced current in the plate, which by its adverse induction, neutralizes the current in the coil. 10. *Currents of different orders.*—Although the secondary current we have been describing consists of merely a wave, enduring but for a moment at the beginning and ending of the primary current, yet it can also produce an induced current, and this in its turn another, and so on. The direction of these currents of the different orders as indicated by the polarity given to a needle in a small coil forming part of the conducting circuit, is as follows:

| | At the beginning. | At the ending. |
|---|---|---|
| Primary current, - - - | + | + |
| Secondary current, - - - | — | + |
| Current of the third order - - | + | — |
| Current of the fourth order, - | — | + |
| Current of the fifth order, - - | + | — |

"From the foregoing table, it ought to be supposed that each succeeding induction consists of a single wave, in the direction indicated by the magnetization of the needle; but this is not the case. The second induced current, for example, at the beginning of the battery current, does indeed consist of a single wave; but since an induced current is produced, at the ending, as well as at the beginning of the inducing current, it follows that the current of the third order must consist principally of two waves, which may be represented by +—; and since each of these will produce in turn two induced waves, the current of the fourth order must be formed of four waves — + + —, and for the same reason the current of the fifth order must consist of eight waves and so on. The first wave in each series is the most intense and hence the magnetic character of the needle is determined by this. Professor Henry has succeeded in referring all these phenomena of galvanic induction to the principle which he has adopted for the explanation of the dynamic induction of ordinary electricity. If we suppose, in the case of a galvanic current, a constant addition of free electricity to one end of the conducting wire, and a constant subtraction of natural electricity from the other, the first end will be *plus*, and the other *minus*, and if, while the wire is in this state, a parallel wire is made to approach it there, according to the laws of statical induction, during the advance of the secondary wire, an induced current of the natural electricity of the wire should be produced in an opposite direction to that of the battery current. When the motion ceases the current must cease. And although the wire is in a state of unusual equilibrium, which may be readily represented by a diagram, it exhibits no signs of excitement so long as the battery current remains the same and the wire is immovable; but if the second wire be drawn back, then its electricity must return to its normal state, and thus an opposite induced current be produced.

"Under the head of galvanic induction we may mention the receat discovery of Dr. Faraday, of the statical inductive influence of magnetism and galvanism on the solid and liquid substances. He divides all bodies belonging to these two states into two classes; one of which is affected by magnetism after the manner of iron, and is called the magnetic class, and the other which is differently affected is denominated the diamagnetic class. When bars of the first class are freely suspended between the legs of a powerful horseshoe magnet, they arrange thomselves, as is well known, in a line passing through the two poles; but when bars of the second class are similarly suspended, they turn into a position at right angles to the same line. Dr. Faraday also finds that a powerful galvanic current passed in a coil around a bar of almost any transparent substance, such as glass, induces on the body a change of molecular arrangement, which is exhibited in the twist given to the plane of polarization of a beam of polarized light transmitted through the substance. The same effect may also be produced by placing the transparent bar between the poles of a powerful magnet. Connected with these indications there is an interesting fact first noticed a few years since by Dr. Page, of the Patent Office, and since studied by a number of persons, of the sound produced in a bar of iron at the magnetization, indicating a change in the molecular arrangement of the metal."

VII. *Animal Electricity.*—In this branch of the subject, the latest researches are so intimately connected with medicine that we offer no apology for the notice of them.

"The most important researches which have recently been made in reference to animal electricity are those of Matteucci, in Italy. The result of his labors conclusively proves, not that the contractions of the muscles are due to a current of electricity from the brain, but that in all living and recently killed animals there exists in the muscle itself a constant current. The general conclusion to which he has arrived in regard to these currents may be briefly stated as follows:—During the whole time the arterial blood is acting on the muscular fibre, a current of electricity is evolved which passes from the fibre to the blood, and thence to the surface, probably the facia of the muscles; or, in other words, the muscular fibre represents the zinc of an ordinary galvanic arrangement, the blood the exciting liquid, and the facia the copper or negative element. Indeed, the development of the electricity in the two cases appears to be due to the same cause, namely, the combination of an oxidizable substance with oxygen. We know the oxygen of the arterial blood acts on every part of the animal economy, and that every part of the organism is undergoing a process of change analagous to combustion, in which carbonic acid is developed, and heat extricated. The electricity in the one case is evolved by the burning of zinc in an acid, and in the other by burning the muscular fibre in arterial blood. For exhibiting these currents, a deep gash may be made transverse to the fibre of a muscle of a recently killed animal; and if one end of the wire of a delicate galvanometer be plunged into the bottom of the gash, care being taken not to touch the sides and the other end of the wire, brought in contact with the facia, the needle will be deflected. By using a series of positions of muscles of recently killed animals, we may form a compound battery; the muscular fibre of one piece being placed in contact with the facia or surface of the other, throughout the series. A pile formed in this way, of twelve or fifteen pieces of eels or muscles of pigeons produces a very marked effect on the galvanometer.

"*Effect of the Electrical Current on the Nerves.*—It is well known that the galvanic current transmitted along a nerve produces contraction in the muscle in which it terminates. According to Matteucci, there are two stages of vitality after the death of the animal, in which the current produces different effects. In the first of these, muscular contractions are produced both at the commencement and interruption of the galvanic current. In the second, they are observed in one case only, either at the beginning or ending of the current. In the case of mixed nerves, or those composed of bundles of nerves of sensation and motion, and which, consequently, have double roots, the contraction, when the current is directed from the centre towards the extremities, is exhibited at the commencement of the current; but with the motor nerves, with a current in the same direction, the contraction is only exhibited at the ending of the current. When the current is in the opposite direction, the phenomena are reversed.

"Some of the researches of Matteucci have a bearing on medical electricity, and he has been led to conclude :—that the electrical current may be used with good success in the treatment of cases of paralysis either partial or total, and also of *tetanus;* but that in the therapeutical application of electricity, we should be careful not to continue the passage of the current too long, lest we augment the disease. The more intense the current the shorter should be its direction. When a current has passed for some time in the same direction through a nerve, paralysis of the nerve ensues; but the sensibility may be almost immediately restored, by passing the current in an opposite direction. In a case of paralysis of the nerve of motion, a current from the extremities to the centre is recommended; for paralysis of sensation, an opposite current must be employed. The most convenient instrument for the application of the electrical current for medical purposes is a single battery coil machine. Some of these instruments are so arranged as to give a series of currents in alternate directions, and others to produce a series of impulses all in the same direction."

When we commenced the above review it was our intention to have given an outline of the article we had selected from the work, whose title heads these pages; but we found, as we progressed, that to condense the already condensed account, would be but to omit portions equally as interesting as others retained. We, accordingly, have extended our notice, perhaps too far, but have to say, in conclusion, that as it now stands, it presents a tolerably perfect view of the present condition of electrical science, a department of physics now so intimate in its connections with medicine, that no medical man should be willing longer to acknowledge himself unacquainted with its principles. J. V. Z. B.

